# Expression of PTEN and p53 and Their Clinicopathological Correlation in Breast Cancer

**DOI:** 10.7759/cureus.101323

**Published:** 2026-01-12

**Authors:** Tonushree Purohit, Soumya Sahu, Manasi Dandekar, Deepshikha Verma

**Affiliations:** 1 Department of Pathology, People's College of Medical Sciences and Research Centre, Bhopal, IND; 2 Department of Pathology, LN Medical College and Research Centre, Bhopal, IND; 3 Department of Pathology, Atal Bihari Vajpayee Government Medical College, Vidisha, IND

**Keywords:** breast carcinoma, immunohistochemistry, p53, prognostic biomarkers, pten

## Abstract

Background: Breast cancer is one of the most common malignancies in women and is a major cause of cancer-related mortality. Alterations in the expression of tumor suppressor genes such as PTEN and p53 may influence tumor behavior and prognosis. This study aimed to evaluate the immunohistochemical expression of PTEN and p53 in breast carcinoma and analyze their association with clinicopathological parameters.

Methods: A cross-sectional study was conducted on 50 histologically confirmed female breast carcinoma cases. Immunohistochemistry (IHC) for PTEN and p53 was performed using standard protocols. PTEN expression was assessed based on cytoplasmic and nuclear staining intensity and categorized as positive or negative. p53 expression was evaluated as nuclear positivity and categorized similarly. Statistical analysis was done using standard significance tests.

Results: The mean patient age was 49.8 years. The most common histologic subtype was infiltrating ductal carcinoma (IDC). Loss of PTEN expression was found in most of the cases and was associated with higher tumor grade and lymph node metastasis. Most cases exhibited p53 overexpression, which showed trends toward an association with higher tumor grade, premenopausal status, and lymph node positivity. An inverse relationship was noted between PTEN loss and p53 positivity.

Conclusions: PTEN loss and p53 overexpression were frequent in breast carcinoma and correlated with aggressive tumor features. Combined assessment of these biomarkers may provide prognostic value and support therapeutic decision-making in breast cancer.

## Introduction

One of the most prevalent cancers in women is breast cancer [1], which has overtaken lung cancer as the leading cause of cancer worldwide [2]. It is a complicated molecular condition brought on by a genetic mutation. One-third to one-quarter of cases of breast cancer are believed to be caused by a hereditary susceptibility gene or genes [3]. For breast cancer treatment to be implemented successfully, it is essential to prioritize raising awareness of the disease's risks, the advantages of early identification, and increased access to care [4,5].

There are several clinicopathological variables, including biomarkers, that indicate a high risk for breast cancer, including tumor size, grade, lymph node status, histologic types, hormone status, and tumor suppressor genes with germline mutations [6,7]. PTEN, a gene whose deletion is known to occur in breast cancer, is situated at chromosome 10q23 and is implicated in carcinogenesis and tumor progression. It contributes to cell proliferation, cell cycle progression, DNA repair, cell adhesion, migration, and differentiation. Uncontrollable tumor growth and aberrant cell cycles are brought on by PTEN mutation [8,9].

The most often altered gene in numerous malignancies, including breast carcinoma, is p53, which is found on chromosome 17p13. It is antiproliferative, aids in angiogenesis, and induces programmed cell death (apoptosis) as a result of genotoxic stress, leading to aberrant cell growth [10]. When clinicopathological aspects of breast cancer patients were correlated with PTEN and p53 expression, the results varied depending on the location and ethnicity. Loss of PTEN and p53 function has been shown to be crucial for the development of breast cancer [11,12].

In this study, in order to better understand therapeutic choices that would reduce mortality and morbidity, we aim to assess the expression of the p53 and PTEN genes in patients with breast cancer and study how clinicopathological factors correlate with them.

## Materials and methods

A cross-sectional study (prospective and retrospective) was conducted in the Department of Pathology, Gandhi Medical College, Bhopal, from 1st January 2021 to 30th June 2022. The Institutional Ethical Committee, Gandhi Medical College, Bhopal, issued approval 27144/MC/IEC/2021. Samples were received as formalin-fixed tissue, and information or history was taken from requisition forms received in the Department of Pathology. The history of cases was also taken through case files and interviews. Histologically diagnosed cases of breast cancer in female patients were included in the present study, while histologically undiagnosed cases, histologically diagnosed cases other than breast carcinomas, and male patients with breast carcinoma and recurrence/patients with neoadjuvant therapy and multiple malignancies were excluded.

Cases reported histopathologically were then selected for immunohistochemistry (IHC) for PTEN and p53 using Pathinsitu kits (PathnSitu Biotechnologies Pvt. Ltd., Telangana, India) for IHC. For IHC assessment, each run of immunostaining, sections of breast cancer tissue with PTEN and p53 positive were used as positive controls, while slides used as negative controls were incubated with phosphate-buffered saline (PBS) in place of the primary antibody. The internal controls for PTEN and p53 expression were the normal epithelial duct and myoepithelial cells [13].

PTEN positivity is analyzed by assessing cytoplasmic and nuclear immunoreactions, which will be scored based on intensity as shown in Table 1 [14].

**Table 1:** Scoring criteria for PTEN immunoreactivity

Score	Intensity	Distribution Pattern	Interpretation
0	Weak	Diffuse, regional, or focal	Negative
1	Moderate	Regional or focal	Negative
2	Moderate	Diffuse	Positive
3	Strong	Diffuse, regional, or focal	Positive

The staining intensity was evaluated by calculating the percentage of positive cells in 100 malignant cells at objective 40, total magnification. p53 positivity will be analyzed by assessing the nuclear positivity as shown in Table 2 [13].

**Table 2:** Scoring criteria for p53 nuclear positivity

Score	Nuclear Reactivity Description	Percentage of Tumor Cells	Interpretation
(-)	No nuclear reactivity	0%	Negative
(+/-)	Few focally positive cells	1% to <10%	Negative
(+)	Heterogeneous nuclear reactivity	10–50%	Positive
(++)	Homogeneous, intense nuclear reactivity	50–100%	Positive

## Results

In our study, we studied 50 diagnosed cases of breast carcinoma. The mean age of the patients in this study came out to be 49.8 years, ranging from 27 to 80 years. Around 28 (56%) cases were below the age of 50 years, while 22 (44%) cases were above the age of 50 years. Out of 50 patients, 28 (56%) are premenopausal, and 22 (44%) are postmenopausal, with ages ranging from 27 to 80 years. Of the patients, 37 (74%) had carcinoma in the right breast, 13 (26%) had carcinoma in the left, and six (12%) had a tumor size less than or equal to 2 cm, 16 (32%) had tumor sizes between 2 and 5 cm, and 28 (56%) had tumor sizes more than 5 cm.

The most common histologic type found in this study was infiltrating ductal carcinoma (IDC). Out of 50 patients, 39 (78%) had IDC type, followed by mucinous carcinoma (5, 10%), invasive lobular carcinoma (3, 6%), and medullary carcinoma (3, 6%). Around 33 (66%) of them had lymph node metastases, compared to 17 (34%) cases of those who did not show metastasis.

Out of 50 patients, 26 (52%) had grade III tumors, 14 (28%) had grade II tumors, and 10 (20%) had grade I tumors (according to the Scarff-Bloom-Richardson grading system), while 40 (80%) patients had stage III, six (12%) had stage II, and four (8%) had stage I (according to the Tumor-Node-Metastasis staging system).

PTEN expression distribution in relation to different histologic types of breast carcinoma

Out of 50 patients, 39 (78%) had IDC type, followed by mucinous carcinoma (5, 10%), invasive lobular carcinoma (3, 6%), and medullary carcinoma (3, 6%). Around 39 (78%) cases were IDC, out of which 20 (51.3%) were negative for PTEN expression, 19 (48.7%) out of them showed weak diffuse staining (score 0), and one (2.6%) showed moderate regional/focal staining (score 1). Around 19 (48.7%) cases were positive for PTEN expression, out of which 10 (25.6%) cases showed moderate diffuse staining (score 2) and nine (23.1%) cases showed strong diffuse staining (score 3).

Out of three (6%) invasive lobular carcinoma cases, two (66.6%) were negative for PTEN expression, with one (33.3%) out of two showing weak diffuse/regional staining (score 0) and one (33.3%) showing moderate regional/focal staining (score 1). One (33.3%) case was positive for PTEN expression and showed moderate diffuse staining (score 2). Out of five (10%) cases of mucinous carcinoma, two (40%) were negative for PTEN expression, all of which showed weak diffuse/regional staining (score 0), and three (60%) cases were positive for PTEN expression and showed strong diffuse staining (score 3). Out of three (6%) cases of medullary carcinoma, two (66.7%) were negative for PTEN expression, all of which showed weak diffuse/regional staining (score 0). One (33.3%) case was positive for PTEN expression and showed strong diffuse staining (score 3). There was no significant statistical correlation between histologic types and PTEN expression.

Out of 50 cases, 10 cases of breast carcinoma had grade I (20%), and three (30%) were negative for PTEN expression and showed weak diffuse staining (score 0). There was no moderate regional/focal staining seen in any of the cases. Seven (70%) cases were positive for PTEN expression, all of them showing strong diffuse staining (score 3). Out of 50 cases, 14 cases are grade II, six (42.9%) were negative for PTEN expression, five (35.7%) out of which showed weak diffuse staining (score 0), and one (7.2%) showed moderate regional/focal staining (score 1). Eight (57.1%) cases were positive for PTEN expression; among them, three (21.4%) cases showed moderate diffuse staining (score 2) and five (35.7%) cases showed strong diffuse staining (score 3). Out of 50 cases, 26 cases were grade III, and 17 (65.4%) were negative for PTEN expression, 16 (61.5%) of which showed weak diffuse staining (score 0), and one (3.9%) showed moderate regional/focal staining (score 1). Nine (34.6%) cases were positive for PTEN expression; among them, eight (30.7%) cases showed moderate diffuse staining (score 2), and one (3.9%) case showed strong diffuse staining (score 3). There is a significant statistical correlation between histologic grade and loss of PTEN expression. The distribution of p53 expression with relation to various histologic types of breast carcinoma is shown in Table 3.

**Table 3:** Distribution of p53 expression with relation to various histologic types of breast carcinoma

	Frequency	Percentage	p53 Expression			
			Positive		Negative	
			++	+	+-	-
Infiltrating ductal carcinoma	39	78%	26 (66.7%)	6 (15.4%)	1 (2.6%)	6 (15.4%)
Invasive lobular carcinoma	03	06%	3 (100%)	0 (0%)	0 (0%)	0 (0%)
Mucinous carcinoma	05	10%	1 (20%)	0 (0%)	1 (20%)	3 (60%)
Medullary carcinoma	03	06%	1 (33.3%)	0 (0%)	1 (33.3%)	1 (33.3%)
Total	50	100%	31	6	3	10

Out of 50 cases, 39 (78%) are IDC, out of which seven (18%) were negative for p53 expression; among them, six (15.4%) showed no nuclear reactivity (-), and one (2.6%) showed focal negative nuclear staining (+-). Around 32 (82.1%) cases were positive for p53 expression; among them, six (15.4%) cases showed heterogeneous nuclear staining (+) and 26 (66.7%) showed homogeneous nuclear staining (++).

All three (6%) invasive lobular carcinoma cases out of 50 cases showed homogenous (100%) nuclear staining (++).

Out of 50 cases, five (10%) were cases of mucinous carcinoma, and four (80%) were negative for p53 expression, out of which three (60%) showed no nuclear reactivity (-) and one (20%) showed focal negative nuclear staining (+-). One (20%) out of five cases was positive for p53 expression and showed homogenous nuclear staining (++).

Out of 50 cases, three (6%) were cases of medullary carcinoma, two (66.7%) were negative for p53 expression, one (33.3%) showed no nuclear reactivity (-), and one (33.3%) showed focal negative nuclear staining (+-). One (33.3%) out of three cases was positive for p53 expression and showed homogenous nuclear staining (++), as shown in Table 4.

**Table 4:** Distribution of PTEN expression in relation to p53 expression Chi-square: 5.88; P-value: <0.05 (0.02)

		p53-Expression		
PTEN expression		Positive	Negative	Total
	Positive	14 (58.3%)	10 (41.7%)	24 (48%)
	Negative	23 (88.5%)	03 (11.5%)	26 (52%)
	Total	37 (74%)	13 (26%)	50

**Figure 1:**
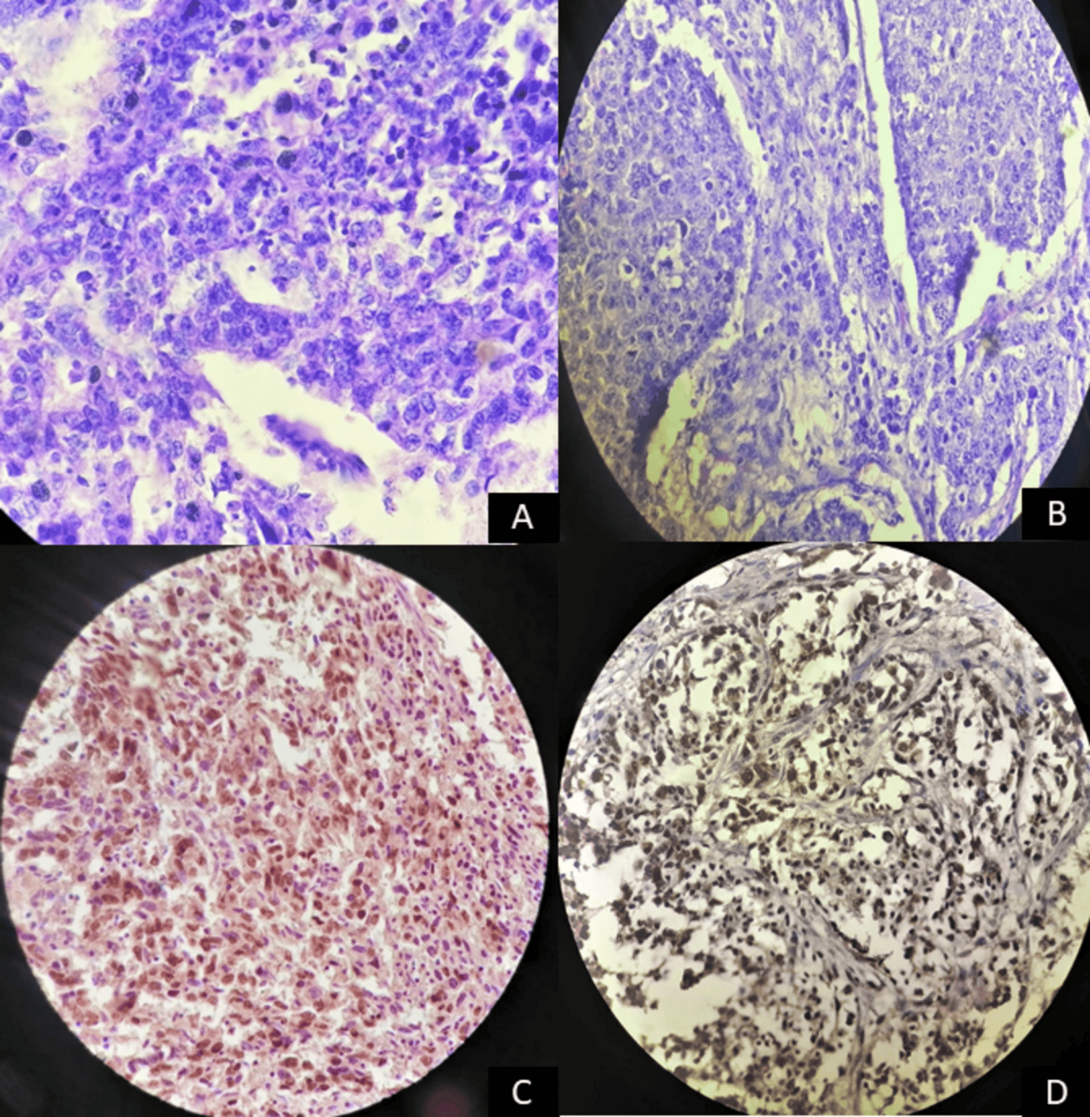
Histological and immunohistochemical image of breast carcinoma A, B: 40X view of invasive ductal carcinoma (H & E stain); C: 40X view of p53 heterogeneous (score +) nuclear staining of infiltrating ductal carcinoma; D: 40X view of PTEN strong diffuse staining (score 3).

In our present study, we found the total PTEN positive to be 24 (48%) out of 50 cases, out of which 14 (58.3%) were positive for p53, and 10 (41.7%) were negative for p53. Out of 50, 26 (52%) cases were PTEN negative, out of which 23 (88.5%) were positive for p53 expression, and three (11.5%) were negative for p53 expression. There was a statistically significant correlation between them. PTEN and p53 positivity is shown in Figure 1.

## Discussion

Breast cancer is the most prevalent cancer among women around the world. The body's genetic alterations lead to this complicated molecular disorder. Its etiology is influenced by both tumor suppressor genes and several genetic alterations. Thanks to fundamental research into cancer, we now have a deeper understanding of the biology and genetics of the disease.

PTEN and p53, two tumor suppressor genes, are essential for regulating cell division and death as well as preventing carcinogenesis, and have a key role in regulating the spread of breast cancer [15]. As a result of tremendous advancements, there is now a far wider range of therapeutic options accessible for the treatment of breast cancer. With additional treatment choices available, it is more important to understand prognostic markers such as age, tumor grade, stage, lymph node status, and hormone receptors, and how they interact with one another [7].

For our study, we collected 50 cases of breast carcinoma (mastectomy specimens) that were received and reported in our tertiary care center. The most common histologic type found in this study was IDC. Out of 50 patients, 39 (78%) had IDC type, followed by mucinous carcinoma (5, 10%), followed by invasive lobular carcinoma and medullary carcinoma (3, 6%). IDC was the most common histologic type observed by Sheikhpour R et al. [16] (84.6%) and Goel et al. [17] (85%) in their separate studies, followed by medullary carcinoma and lobular carcinoma.

Immunohistochemical expression of PTEN and p53

Immunohistochemical expression of PTEN and p53 was studied in 50 cases of breast carcinoma, and its expression was compared with clinical variables like age, size of the tumor, and menstrual status, and pathological variables like histological grade, lymph node status, stage of the tumor, and estrogen receptor (ER), progesterone receptor (PR), and human epidermal growth factor receptor 2 (HER2)/neu status.

We also compared the expression of PTEN with p53. The comparison between immunohistochemical expression of PTEN and p53 with various clinicopathological variables is aimed at arriving at a relationship between these variables and indirectly aiding in the management, risk stratification, and outcome of the patient. PTEN expression loss was more common in people older than 50 years (73.1%), according to AW Al-bideri et al. [13] (n=117) (p=0.05). Age and loss of PTEN expression were not shown to be significantly correlated in another study done by Chang et al. [18]. In the present study, 57.1% showed loss of PTEN (score 0,1) expression in ages less than 50 years, and 45.4% showed loss of PTEN expression in ages more than 50 years. In the present study, no significant statistical correlation was found between age and PTEN expression (p=0.3).

Genes like BRCA1 and BRCA2 enhance the risk of hereditary breast cancer even at a young age, and genetic factors contribute to the development of breast cancer at a younger age. The expression silencing of the PTEN protein may also be significantly impacted by the PTEN gene mutation and other mechanisms, such as promoter methylation, translational, and post-translational regulation [18].

Salmani H et al. [2] studied the relationship between clinicopathological factors and PTEN expression in 100 samples and found 28.9% of IDC showed loss of PTEN expression, and other histologic types showed low PTEN expression, i.e., 5.3%, and there was no correlation between them. Similar results were found in a study done by AW Al-bideri et al. [13], where 62.7% of IDC showed loss of PTEN expression, 60% of lobular carcinoma, and 80% of medullary carcinoma showed loss of PTEN expression (p=0.5).

In the present study, IDC was the most common histologic type and showed 51.3% loss of PTEN expression, followed by mucinous carcinoma, medullary carcinoma, and invasive lobular carcinoma, showing 40%, 66.7%, and 66.6%, respectively. There was no correlation found between them (p-value=0.5). This might be as a result of our small sample size and the insignificant number of various breast cancer subtypes [7]. According to AW Al-bideri et al., there was a significant difference (P=0.05) in the decrease of PTEN expression between lymph node-positive and lymph node-negative cases of breast cancer [13]. A further study by Chang et al. [18] reported a substantial correlation between reduced expression of the PTEN gene and lymph node metastasis in patients with breast cancer (p=0.04). According to Zhang et al. [19], there was no significant association (p-value=0.6) between the presence of lymph node metastases and the reduction of PTEN gene expression.

In the present study, PTEN loss of expression was seen in 54.6% of lymph node-positive cases, whereas 47.1% was seen in lymph node-negative cases. There was a significant correlation found between lymph node metastasis and loss of PTEN expression (p-value=0.02). This signifies that PTEN protein loss is much higher in tumors with positive lymph node status and might contribute to the progression and aggressiveness of breast cancer, and is directly related to poor prognosis. Salmani H et al. [2] studied histologic types (n=78) and p53 expression and found that IDC showed 52.6% high p53 expression and other carcinomas showed 14.5% high p53 expression. No significant correlation was found between histologic type and high p53 expression (p-value=0.7).

According to a study done by AW Al-bideri et al. [13], 67% of IDC showed high p53 expression, whereas lobular carcinoma and medullary carcinoma showed 42.9% and 69.2% high p53 expression, respectively. No significant correlation was found between histologic type and p53 expression. (p=0.5) Similar results were found by a study conducted by Sheikhpour R et al. [16]; 86.6% of IDC showed high p53 expression, and other carcinomas, i.e., 6.6%, showed high p53 expression. No significant correlation was found between the histologic type and p53 expression (p-value=1.0).

A study conducted by Song HS et al. [20] also concluded that there was no significant correlation between histologic type and p53 high expression (p-value=0.14). In the present study, the most common histologic type was found to be IDC, i.e., 82.1% showed high p53 expression. ILC showed 100% high p53 expression. Mucinous carcinoma showed 20%, and medullary carcinoma showed 33.3% high p53 expression. (p-value=0.5) There was no statistically significant association between high p53 expression and histologic type. This may be due to our study's small sample size and lack of a substantial number of distinct breast cancer subtypes [7].

In the study conducted by AW Al-bideri et al. [13] (n=117), there was no significant correlation found between the two markers. Around 83 cases were found to be p53 positive; 28 (33.7%) of them were positive for PTEN, and 55 (66.3%) cases were negative for PTEN. Seventy-six cases were PTEN negative; 55 (72.4%) were positive for p53, and 21 (27.6%) cases were negative for p53 (p=0.2). In our present study, we found the total PTEN positive to be 24 (48%) out of 50 cases, out of which 14 (58.3%) were positive for p53, and 10 (41.7%) were negative for p53. Thirty-seven cases were p53 positive, out of which 14 (58.3%) were positive for PTEN and 23 (88.5%) were negative for PTEN. There was a statistically significant correlation between them (p-value=0.02).

By restricting Mdm2 in the cytoplasm and promoting destruction, PTEN shields wild p53 protein from Mdm2 degradation; however, loss of PTEN function may result in Mdm2 protein degrading p53 more quickly, losing its function, and accelerating carcinogenesis [19].

Limitations

This study has several limitations. First, the sample size was relatively small, which may have reduced the statistical power and limited the ability to detect significant associations between biomarker expression and clinicopathological variables. The small representation of less common breast cancer subtypes further restricted subtype-based comparisons. Second, the study was conducted at a single center, which may limit the generalizability of the findings to wider populations. Third, only immunohistochemical evaluation was performed; molecular testing, such as gene sequencing or methylation analysis, was not included, which could have provided deeper insights into the mechanisms underlying PTEN and p53 alterations.

## Conclusions

As this study was conducted in a hospital, it might not accurately reflect the disease's actual prevalence in the population. Changes in the p53 gene result in the absence of this negative growth control and faster cell proliferation. The fraction of p53-positive cancer cells seems to be closely correlated with the aggressiveness of breast cancer. Breast cancer frequently experiences PTEN loss, which is strongly linked to quicker progression and a worse prognosis.

In our study, immunohistochemical expression showing loss of PTEN was significantly associated with clinicopathological variables like high tumor size, lymph node metastasis, and high grade of tumor. Similar results were seen in previous studies. High expression of p53 was associated with high-grade tumors, lymph node metastasis, age less than 50 years, and premenopausal status, showing poor prognosis and progression of breast carcinoma, which was in concordance with other studies. We also studied the relationship between PTEN and p53. By restricting Mdm2 in the cytoplasm and promoting destruction, PTEN shields wild p53 protein from Mdm2 degradation. However, when the PTEN function is lost, the Mdm2 protein may degrade p53 more quickly, losing its function and encouraging carcinogenesis. Understanding the prognostic relevance of the markers and their role can assist in developing novel treatment options by improving the way the disease is approached.
